# Investigation of the STR loci noise distributions of PowerSeq™ Auto System

**DOI:** 10.3325/cmj.2017.58.214

**Published:** 2017-06

**Authors:** Xiangpei Zeng, Jonathan L. King, Bruce Budowle

**Affiliations:** 1 Center for Human Identification, University of North Texas Health Science Center, Fort Worth, TX, USA; 2 Center of Excellence in Genomic Medicine Research (CEGMR), King Abdulaziz University, Jeddah, Saudi Arabia

## Abstract

**Aim:**

To characterize the noise and stutter distribution of 23 short tandem repeats (STRs) included in the PowerSeq^TM^ Auto System.

**Methods:**

Raw FASTQ files were analyzed using STRait Razor v2s to display alleles and coverage. The sequence noise was divided into several categories: noise at allele position, noise at -1 repeat position, and artifact. The average relative percentages of locus coverage for each noise, stutter, and allele were calculated from the samples used for this locus noise analysis.

**Results:**

Stutter products could be routinely observed at the -2 repeat position, -1 repeat position, and +1 repeat position of alleles. Sequence noise at the allele position ranged from 10.22% to 28.81% of the total locus coverage. At the allele position, individual noise reads were relatively low.

**Conclusion:**

The data indicate that noise generally will be low. In addition, the PowerSeq^TM^ Auto System could capture nine flanking region single nucleotide polymorphisms (SNPs) that would not be observed by other current kits for massively parallel sequencing (MPS) of STRs.

Capillary electrophoresis (CE)-based technology has been the primary methodology to analyze short tandem repeats (STRs) in forensic DNA human identification testing for the past two decades. With the development of massively parallel sequencing (MPS), another viable platform is available for typing STRs. Several studies already have revealed the potential value of MPS for STR typing (1-16). MPS technology enables characterization of a locus based on sequence instead of length-based differences as in CE, so it can increase the discrimination power of some STRs (2,9). MPS is able to detect repeat motif variation (RMV) within STRs, and single nucleotide polymorphisms (SNPs) residing within repeats and within the flanking regions.

However, use of MPS technology also brings different challenges. Sequence data likely will assist in resolving mixture evidence better than CE-based data. There are two types of noise that must be addressed in order to develop meaningful guidelines for mixture interpretation, especially for trace level contributors. First, stutter, ie, slippage events during PCR, is well-defined (17-19) and is inherent in STR typing. Typically, -1 and less often +1 stutter are observed in CE-generated data. Other stutter artifacts (eg, -2, -3, and so on) are not observed because the signal for these less frequently occurring artifacts are buried within the noise. With MPS multiple stutter products can be observed especially when read depth (or coverage) is exceedingly high. Second, there is sequence noise due to a low-level sequence substitution (SSE) and/or insertion/deletion error (IDE) rate. Such artifacts may exist with CE-based data as well but again often is not observed because it cannot be distinguished from background noise. However, MPS allows for detection of each molecule (or in actuality each molecular clone). These artifact features of STR typing with MPS must be described and defined per locus (20,21) to establish minimum thresholds and/or probabilities of events for an effective mixture interpretation protocol for MPS data.

Using data from Zeng et al (14), the noise and stutter distribution were characterized for 23 STRs included in PowerSeq^TM^ Auto System (CSF1PO, D10S1248, D12S391, D13S317, D16S539, D18S51, D19S433, D1S1656, D21S11, D22S1045, D2S1338, D2S441, D3S1358, D5S818, D7S820, D8S1179, DYS391, FGA, Penta D, Penta E, TH01, TPOX, and vWA) (Promega Corporation, Madison, WI, USA). While the sample size is small (as this is a preliminary study to describe the main artifacts), the results show that multiple stutter species can be observed. Moreover, sequence noise, which can be a reasonably large component of the total reads, is comprised of many different species such that the actual maximum sequence noise threshold per species for most STRs is very low. In addition to the artifact study, flanking SNPs were identified that would not be detected in other MPS commercial kits due to primer placement, emphasizing the point that population data for some STR haplotype variants will be kit-specific.

## METHODS AND MATERIAL

The samples, extraction, PCR amplification, library preparation, and sequencing are described in Zeng et al (14).

Raw FASTQ files were exported from the MiSeq instrument and analyzed using STRaitRazor v2s (22) to display alleles and coverage. For each sample, only the homozygous loci and heterozygous loci with alleles at least four repeats difference in size were used in the noise data analysis, so that extended stutter products could be observed unequivocally. The sequence noise was divided into several categories as follows: noise at allele position, noise at -1 repeat position, and artifact. The artifacts include noise at positions<-2 repeat and>+1 repeat positions, and incomplete variants (ie, IDE) between -2 repeat and +1 repeat positions. For simplicity of presentation, stutter and sequence noise with the same nominal length of stutter were combined into stutter at -2 repeat and +1 repeat positions. For heterozygotes, the noise, stutter, and allele reads of two alleles were combined and treated as homozygotes. For each sample at each STR locus, the reads of sequence noise, stutter, and allele were divided by the locus coverage to obtain the relative percentages of locus coverage. Finally, the average relative percentages of locus coverage for each noise, stutter, and allele were calculated from the samples used for this locus noise analysis.

### Flanking region and repeat region SNPs

Maximum haplotype coordinates for the 21 overlapping loci were identified between 3 MPS systems, ie, Promega PowerSeq™ Auto System, ForenSeq™ DNA Signature Prep Kit (Illumina, San Diego, CA, USA), and Precision ID GlobalFiler^TM^ Mixture ID panel (Thermo Fisher Scientific, South San Francisco, CA, USA), as well as Penta D and Penta E for a total of 23 STR loci. BED files were converted to HG19 and variants were identified within these regions using UCSC’s Table Browser (23). Variant positions were further reduced to remove STRs and SNPs and insertion/deletions (InDel) with allele frequencies below 0.05 in all super populations of the 1000 Genomes Project (24,25).

## RESULTS AND DISCUSSION

In this study, the noise distributions of 23 STRs of the PowerSeq^TM^ Auto System were investigated. The STR locus genotype, locus coverage, and the number of samples used for each STR noise analysis are shown in Table 1. The number of samples used in the data analysis ranged between 2 and 11. For example, for the CSF1PO locus, seven samples could be used in the stutter and noise analysis; all were homozygotes.

**Table 1:** The short tandem repeat (STR) loci genotypes and coverage (in parentheses) of the 14 samples that could be used in the noise distribution analysis

	Samples														
STR locus	025	008	393	011	047	SB10	29640	07593	72641	39763	00214	39720	07851	91147	No. of samples used
CSF1PO	12(2302X)	12(2145X)	12(3111X)						10(1693X)		11(818X)	11(1782X)		10(1321X)	7
D10S1248				13 (3745X)	13,17 (17801X)		13(11017X)			13(1184X)	13,17(782X)		13(4517X)	14(1606X)	7
D12S391	16,22 (1422X)					18,23 (920X)	17,22 (10263X)				19,23 (1287X)				4
D13S317						8,13 (1380X)			12(1466X)			11(1342X)			3
D16S539	11(988X)									11(1373X)					2
D18S51	12,17 (1328X)	14(2083X)	14,18 (4823X)		12,16 (8624X)								17(3250X)		5
D19S433		14(2082X)			14(9989X)				15(1067X)		15(827X)				4
D1S1656			13,17.3 (3501X)				13(6098X)	12,17.3 (5240X)		11,15 (1332X)			11,15.3 (3633X)		5
D21S11		27,31 (2538X)									27,32.2 (866X)				2
D22S1045	15(1770X)	11,15 (5839X)	11,16 (3465X)	15 (3205X)	15(15335X)	15 (1606X)	11,15 (13991X)			15 (1872X)		11(2203X)	16(3306X)	16(1875X)	11
D2S1338	17,23 (1369X)	17,23 (2584X)	18,25 (3133X)		17,23 (9880X)		17,24 (5677X)	18,25(4819X)	17,22 (1919X)	19,23 (1330X)		16,25 (1678X)	17,25 (2686X)		10
D2S441			10,15 (2694X)		11(12133X)	11(875X)				10,14 (1241X)	10,14 (1017X)			11(943X)	6
D3S1358	16(923X)		14,18 (2038X)							15(955X)		15(1429X)			4
D5S818											12(892X)			12(1352X)	2
D7S820		8,12 (1133X)	11(3386X)		12(12472X)									11(1454X)	4
D8S1179	13(826X)		13(2537X)											12(852X)	3
DYS391				9(2024X)	10(8809X)	11(691X)	10(6582X)	10(3320X)						10(990X)	6
FGA		20,24 (2245X)				22,26 (1324X)	22(7254X)								3
Penta D									9,13(1563X)	9,14(1395X)				10(1088X)	3
Penta E	7,12 (850X)	11,15 (1871X)		12(1790X)		7(1255X)	7,14(6961X)	7,15(5393X)	5,17(1138X)	5,15(982X)			7,19 (2146X)		9
TH01		6(2688X)						9.3(4921X)			9.3(812X)	9.3(1116X)		6(1138X)	5
TPOX							8(11289X)					8(1649X)			2
vWA							17(6558X)		17(1422X)		14,18 (563X)	17(1444X)		17(1293X)	5

**Figure 1:**
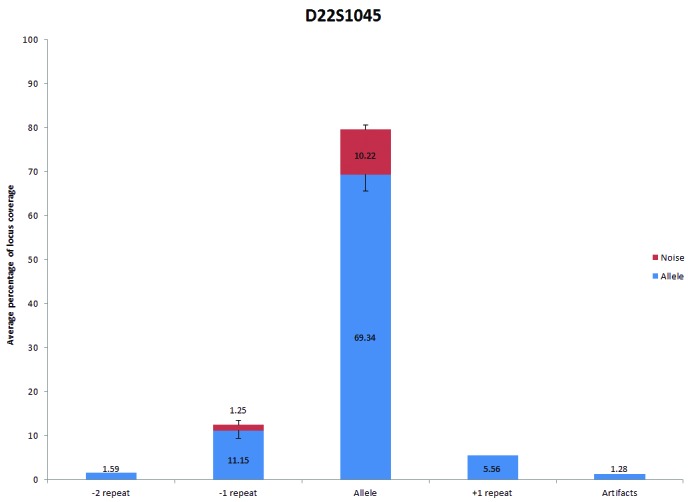
The average percentage of locus coverage for noise, stutter, and allele of the D22S1045 locus in eleven samples. At allele and -1 repeat positions, the upper error bar is standard deviation of noise reads, and the lower error bar is standard deviation of allele or stutter reads.

**Figure 2:**
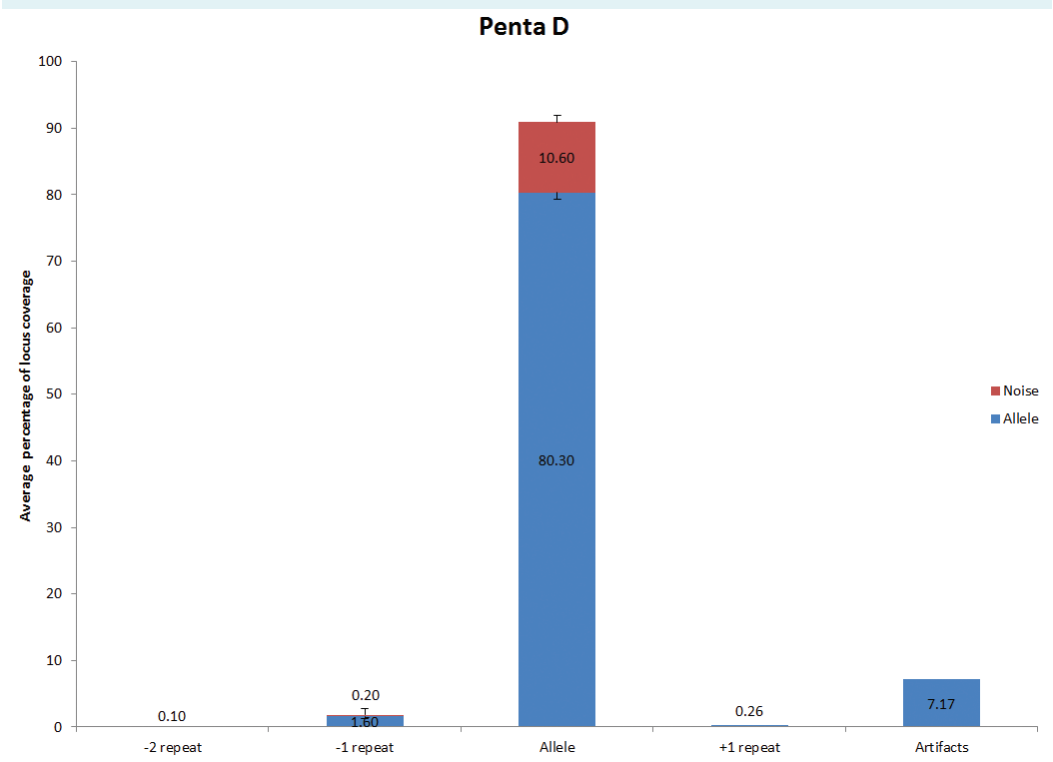
The average percentage of locus coverage for noise, stutter, and allele of the Penta D locus in three samples. At allele and -1 repeat positions, the upper error bar is standard deviation of noise read, and the lower error bar is standard deviation of allele or stutter reads.

**Figure 3:**
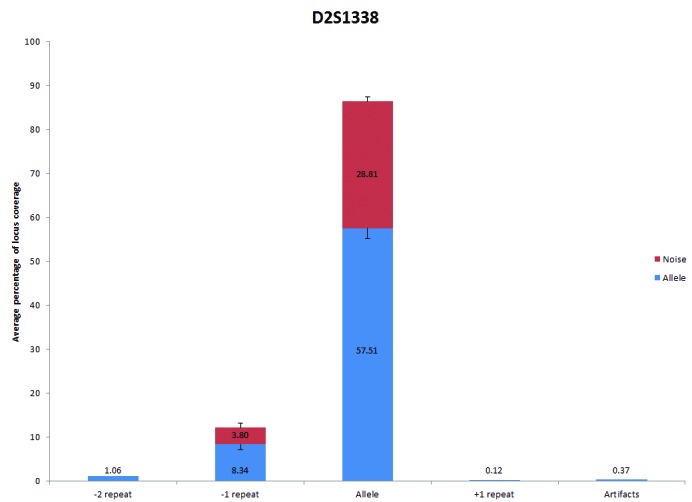
The average percentages of locus coverage for noise, stutter, and allele of the D2S1338 locus in ten samples. At allele and -1 repeat positions, the upper error bar is standard deviation of noise reads, and the lower error bar is standard deviation of allele or stutter reads.

The noise distributions of 23 STRs are shown in Table 2. The D22S1045 and Penta D loci had relatively low noise percentages (10.22% and 10.60%, respectively) at the allele position. But the D22S1045 locus had high levels of stutter at the -1 repeat (11.15%) and +1 repeat (5.56%) positions (Figure 1). For the Penta D locus, stutter and noise were 1.60% and 0.20% of the total locus coverage at the -1 repeat position, respectively (Figure 2). For -2 repeat and +1 repeat stutter positions, the percentages of reads were 0.10% and 0.26%, respectively, of the total locus coverage. However, the Penta D locus had the second highest level artifacts (7.17%, only lower than the D7S820 locus), such as IDEs. In contrast, the D2S1338 locus had the highest percentage of noise (28.81%) of the multiplex at the allele position (Figure 3). At the -1 repeat stutter position, stutter and noise were 8.34% and 3.80%, respectively, of the total locus coverage. For -2 repeat and +1 repeat stutter positions, the percentages of reads were 1.06% and 0.12%, respectively.

**Table 2:** The average percentages of locus coverage for noise, stutter, and allele of 23 short tandem repeats (STRs) of the PowerSeq^TM^ Auto System*

	Average %				
STR locus	N-2 repeat stutter	N-1 repeat (stutter/noise)	Allele (allele/noise)	N+1 repeat stutter	Artifacts
CSF1PO	0.49	6.57/1.26	74.06/16.23	0.76	0.65
D10S1248	1.17	8.64/1.97	68.59/18.67	0.60	0.50
D12S391	1.50	12.25/2.80	65.38/17.30	0.20	0.70
D13S317	0.37	5.70/1.67	70.70/19.87	1.17	0.50
D16S539	1.15	7.15/2.75	69.25/17.95	1.20	0.50
D18S51	1.12	8.98/3.24	63.34/22.38	0.46	0.62
D19S433	1.48	8.73/3.85	56.23/28.33	0.70	0.60
D1S1656	1.16	9.12/1.80	68.82/14.28	1.46	3.47
D21S11	0.85	6.15/3.20	65.20/22.55	1.85	0.70
D22S1045	1.59	11.15/1.25	69.34/10.22	5.56	1.28
D2S1338	1.06	8.34/3.80	57.57/28.81	0.12	0.37
D2S441	0.37	4.83/1.30	73.57/17.05	2.72	0.21
D3S1358	0.78	9.63/2.35	65.73/20.50	0.50	0.51
D5S818	0.50	8.50/1.20	74.55/14.00	1.10	0.20
D7S820	0.68	6.65/1.65	64.08/17.35	0.45	9.11
D8S1179	0.87	8.30/1.73	72.53/15.23	1.13	0.17
DYS391	0.76	6.61/2.30	73.85/15.36	0.24	0.92
FGA	0.93	8.30/2.20	64.17/21.73	2.03	0.62
Penta D	0.10	1.60/0.20	80.30/10.60	0.26	7.17
Penta E	0.16	3.98/0.99	76.01/18.19	0.38	0.36
TH01	0.18	2.38/1.76	67.12/27.54	0.10	1.06
TPOX	0.25	2.40/0.90	74.60/21.05	0.20	0.47
vWA	1.34	7.78/3.50	64.92/21.64	0.10	0.72

*At allele and N-1 repeat positions, the percentages of noise reads were calculated separately from allele/stutter reads. The artifacts include noise at positions<N-2 repeat,>N+1 repeat, and incomplete variants (ie, IDE) between N-2 repeat and N+1 positions.

**Figure 4:**
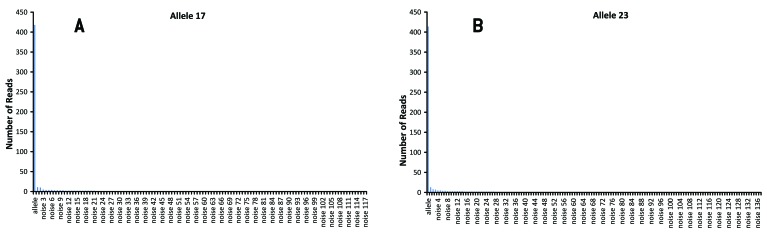
Distribution of sequence noise species and counts at the true allele positions of the D2S1338 locus for sample 025. Figure 4A – allele 17. Figure 4B – allele 23.

The distribution of the sequence noise at the allele position of the D2S1338 locus is shown for one sample (No. 025; genotype 17, 23) to show the range of sequence noise variation and magnitude of any single species associated with an allele (Figure 4). Other than reads that were the attributed to the true allele (ie, the same sequence with the most abundant reads), there were 117 and 137 sequence noise species that were the same length as alleles 17 and 23, respectively. Although combined, the noise species were 12% and 15% of the total reads at this locus for sample 025, the highest individual sequence noise reads were 11X and 13X for allele 17 (418X) and allele 23 (414X), respectively. The majority of noise species had only 1X coverage. Most of the noise likely are due to SSE and are chemistry related. But the overall low level of individual noise species indicates that most SSE are low and thresholds may be set relatively low (ie, well below the total noise observed in this study of 28.81% for the D2S1338 locus). Alternatively, since the noise may be characteristic of an allele at a locus, it may be possible to use the species distribution to resolve contributors of a mixture, making sequence data even more robust for mixture interpretation.

**Figure 5:**
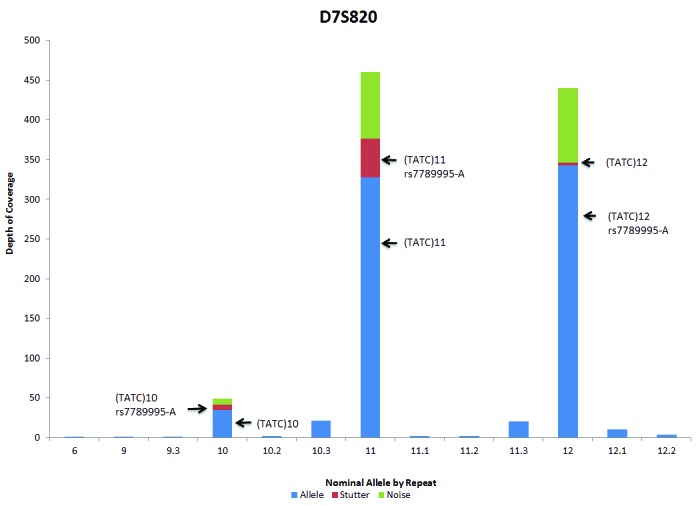
The sequence results of the D7S820 locus for sample 025. The genotype is 11, 12. The repeat motif of allele 12 is (TATC)_12_ and a flanking region SNP (rs7789995 T→A). Based on this flanking region SNP, the stutters from allele 12 are identified at the position of allele 11. The repeat motif of allele 11 is (TATC)_11_ but the flanking region SNP site contains a T. The stutter from allele 11 is detected at the position of allele 12. At position 10 the reads are a combination of two stutters (from allele 11 and allele 12) and sequence noise.

A total of 150 SNPs were identified in the maximum haplotype region of the 23 STR loci. The SNPs frequencies in five populations were downloaded from 1000 Genomes Project (24,25). SNPs with frequency ≥0.05 in at least one of five major populations (African, Ad Mixed American, East Asian, European, and South Asian) were selected. Thirty-four SNPs (19 flanking region and 15 repeat region SNPs) were identified within the STR haplotypes included in the PowerSeq^TM^ Auto System (Table 3). Compared with ForenSeq^TM^ DNA Signature Prep Kit and Precision ID GlobalFiler Mixture ID panel (in-house data, data not shown), there were nine (seven unlinked) flanking region SNPs that were identified only in the PowerSeq STR amplicons. While detection of SNPs within repeats of alleles and corresponding stutter products may be affected by slippage, flanking region SNPs are far more stable and may be extremely useful for resolving stutter from minor or trace contributor alleles of the same nominal length in a mixture. Figure 5 demonstrates the possible application of flanking region SNPs. The genotype of sample No. 025 is 11, 12 at the D7S820 locus. The repeat motif of allele 11 is (TATC)_11_ and the haplotype for allele 12 is (TATC)_12_ with a flanking region SNP (rs7789995 T→A). Based on this flanking region SNP, the stutters from allele 12 are readily identified at the positions of allele 11 and stutter 10. In addition, the stutters caused by allele 11 also are detected at allele position 12 and stutter position 10.

**Table 3:** The short tandem repeat (STR) locus and flanking region-single nucleotide polymorphisms (SNPs) that could be captured by PowerSeq^TM^ Auto System (rs11063970 and rs11063971 are linked.)*

SNP	Region	Chromosome	Position	STR locus	PowerSeq	ForenSeq	MixtureID	REF	ALT	AMR	AFR	EAS	EUR	SAS
rs563636310	Flanking	chr10	129294243	D10S1248	+	+	+	T	A	0.05	0.05	0.04	0.03	0.05
rs77312049	Repeat	chr12	12297095	D12S391	+	+	+	T	C	0.17	0.09	0.13	0.28	0.27
rs73525369^†^	Flanking	chr13	82147972	D13S317	+	-	-	G	A	0.01	0.06	-	-	-
rs9546005	Flanking	chr13	82148069	D13S317	+	+	+	A	T	0.35	0.38	0.49	0.41	0.48
rs202043589	Flanking	chr13	82148073	D13S317	+	+	+	A	T	0.07	0.07	0.06	0.06	0.05
rs1728369^†^	Flanking	chr16	86352607	D16S539	+	-	-	A	C	0.16	0.45	0.23	0.18	0.20
rs11642858	Flanking	chr16	86352761	D16S539	+	+	-	A	C	0.32	0.24	0.44	0.09	0.24
rs147936416	Repeat	chr19	29926289	D19S433	+	+	+	TTC	T	0.19	0.23	0.33	0.10	0.22
rs78443572	Repeat	chr1	230769616	D1S1656	+	+	+	C	T	0.18	0.33	0.19	0.19	0.46
rs141376519	Repeat	chr1	230769665	D1S1656	+	+	+	CT	C	0.26	0.18	0.11	0.28	0.07
rs4847015	Flanking	chr1	230769689	D1S1656	+	-	+	C	T	0.31	0.18	0.12	0.34	0.08
rs13049099	Repeat	chr21	19181992	D21S11	+	+	+	G	A	0.41	0.32	0.58	0.38	0.40
rs6736691	Flanking	chr2	218014824	D2S1338	+	-	+	C	A	0.28	0.19	0.14	0.33	0.11
rs9678338	Repeat	chr2	218014870	D2S1338	+	+	+	C	A	0.63	0.54	0.56	0.62	0.50
rs6736805	Repeat	chr2	218014925	D2S1338	+	+	+	C	A	0.31	0.35	0.22	0.37	0.24
rs62182233	Repeat	chr2	218014929	D2S1338	+	+	+	C	A	0.01	0.08	0.01	0.00	0.06
rs74640515	Flanking	chr2	68011922	D2S441	+	+	+	G	A	0.05	-	0.11	0.03	0.07
rs200211877	Repeat	chr2	68011964	D2S441	+	+	+	CT	C	0.04	0.04	0.07	0.07	0.06
rs13019438	Repeat	chr2	68011990	D2S441	+	+	+	A	G	0.34	0.08	0.16	0.12	0.30
rs2624663	Repeat	chr3	45540750	D3S1358	+	+	+	A	G	0.83	0.49	0.86	0.80	0.84
rs71325067	Repeat	chr3	45540754	D3S1358	+	+	+	A	G	0.22	0.16	0.18	0.27	0.30
rs25768^†^	Flanking	chr5	123775612	D5S818	+	-	-	A	G	0.83	0.82	0.95	0.73	0.89
rs7786079^†^	Flanking	chr7	84160161	D7S820	+	-	-	A	C	0.04	0.19	0.03	0.01	0.08
rs7789995	Flanking	chr7	84160204	D7S820	+	+	-	T	A	0.91	0.99	0.94	0.87	0.92
rs16887642	Flanking	chr7	84160286	D7S820	+	+	+	G	A	0.05	0.18	0.20	0.07	0.16
rs111782616	Repeat	chr8	124894876	D8S1179	+	+	+	A	G	0.14	0.35	0.23	0.12	0.22
rs542851842	Repeat	chr8	124894880	D8S1179	+	+	+	A	G	0.01	0.10	-	0.00	0.00
rs79373318^†^	Flanking	chr11	2171244	TH01	+	-	-	C	T	0.01	0.11	-	-	-
rs13422969^†^	Flanking	chr2	1489544	TPOX	+	-	-	C	A	0.01	0.19	-	0.00	-
rs75219269	Flanking	chr12	5983970	vWA	+	+	+	A	G	0.08	0.03	0.25	0.13	0.15
rs74980505	Repeat	chr12	5984033	vWA	+	+	+	C	T	0.06	0.03	0.25	0.12	0.14
rs11063969^†^	Flanking	chr12	5984116	vWA	+	-	-	A	T	0.05	0.02	0.24	0.11	0.14
rs11063970^†^	Flanking	chr12	5984121	vWA	+	-	-	C	T	0.05	0.02	0.24	0.11	0.14
rs11063971^†^	Flanking	chr12	5984134	vWA	+	-	-	T	C	0.05	0.02	0.24	0.11	0.14

*Abbreviations: REF is the reference allele of the SNP, and ALT is the alternate allele. AFR: African, AMR: Ad Mixed American, EAS: East Asian, EUR: European, SAS: South Asian

†SNPs detected solely by the amplicons in PowerSeq^TM^ Auto System.

### Conclusion

In this study, the stutter, sequence noise distribution, and potential detection of additional flanking region SNPs of the 23 STRs included in PowerSeq^TM^ Auto System were investigated. Stutter products could be observed readily at 2 repeats less and 1 repeat greater than the true allele. Total sequence noise at the allele position ranged from a low of 10.22% to a high of 28.81% of the total locus coverage. However, individual noise species were relatively low indicating that for most STRs noise likely will not have a substantial negative impact on mixture interpretation. Because of primer positioning, the PowerSeq^TM^ Auto System could capture nine (seven unlinked) flanking region SNPs that would not be observed by both the ForenSeq^TM^ DNA Signature Prep Kit and the Precision ID GlobalFiler^TM^ Mixture ID panel. Thus, some STR haplotype allele variation will be multiplex specific.
